# Assessing the impact of ambient temperature on the risk of hand, foot, and mouth disease in Guangdong, China: New insight from the disease severity and burden

**DOI:** 10.1371/journal.pntd.0010470

**Published:** 2022-06-23

**Authors:** Zhicheng Du, Wangjian Zhang, Shicheng Yu, Shao Lin, Yuantao Hao

**Affiliations:** 1 Department of Medical Statistics, School of Public Health, Sun Yat-sen University, Guangzhou, China; 2 Center for Health Information Research, Sun Yat-sen University, Guangzhou, China; 3 Sun Yat-sen Global Health Institute, Sun Yat-sen University, Guangzhou, China; 4 Chinese Center for Disease Control and Prevention, Beijing, China; 5 Department of Environmental Health Sciences, School of Public Health, University at Albany, the State University of New York, Albany, New York, USA; 6 Peking University Center for Public Health and Epidemic Preparedness & Response, Beijing, China; Chengde Medical University, CHINA

## Abstract

**Background:**

The association between the incidence of hand, foot, and mouth disease (HFMD) and ambient temperature has been well documented. Although the severity of symptoms is an important indicator of disease burden and varies significantly across cases, it usually was ignored in previous studies, potentially leading to biased estimates of the health impact of temperature.

**Methods:**

We estimated the disability-adjusted life year (DALY) by considering the severity of symptoms for each HFMD case reported during 2010–2012 in Guangdong and used distributed lag-nonlinear models to estimate the association between the daily average temperature and daily DALY of HFMD cases at the city-level. We investigated the potential effect modifiers on the pathway between temperature and DALY and pooled city-specific estimates to a provincial association using a meta-regression. The overall impact of temperature was further evaluated by estimates of DALYs that could be attributed to HFMD.

**Results:**

The overall cumulative effect of daily mean temperature on the DALY of HFMD showed an inverse-U shape, with the maximum effect estimated to be *β* = 0.0331 (95%CI: 0.0199–0.0463) DALY at 23.8°C. Overall, a total of 6.432 (95%CI: 3.942–8.885) DALYs (attributable fraction = 2.721%, 95%CI: 1.660–3.759%) could be attributed to temperature exposure. All the demographic subgroups had a similar trend as the main analysis, while the magnitude of the peak of the temperature impact tended to be higher among the males, those aged ≥3yrs or from the Pear-River Delta region. Additionally, the impact of temperature on DALY elevated significantly with the increasing population density, per capita GDP, and per capita green space in parks.

**Conclusions:**

Temperature exposure was associated with increased burden of HFMD nonlinearly, with certain groups such as boys and those from areas with greater population density being more vulnerable.

## Author summary

Hand, foot, and mouth disease (HFMD) is a contagious viral infection common in young children. Although the association between ambient temperature and the risk of HFMD has been well documented, existing studies were solely based on the reported number of cases, failing to differentiate cases of different severity. This study improves the estimation of disease burden by additionally accounting for the severity of symptoms. Our major findings included that (1) we identified an inverse-U shaped association between the ambient temperature and the daily DALY (i.e. an established indicator for disease burden) associated with HFMD, with 2.7% DALY could be attributed to the temperature exposure; (2) the peak impact of temperature on DALY was estimated to be *β* = 0.0331 (95%CI: 0.0199–0.0463) DALY and identified at 23.8°C; (3) the trends were generally consistent across different demographic and geographic subgroups, with the peak effect identified to be greater among the males, those aged ≥3yrs or from the Pear-River Delta region; (4) the impact of temperature on DALY elevated more apparently in areas with a greater population density, per capita GDP, or per capita green space in parks.

## 1. Introduction

Hand, foot, and mouth disease (HFMD) is a common infection among children under 5 years old [1]. It is a very contagious condition [1], with large outbreaks reported frequently in multiple Asian countries [2–5]. China has more than 2 million cases being reported annually, making it a country suffering from the heaviest burden of HFMD worldwide, especially in southern regions such as Guangdong [6]. The severity of symptoms is varying substantially across individuals, with most cases characterized by typical and mild clinical manifestations and recovering in a few days without specific medical care [7, 8]. In contrast, some cases may suffer from severe complications which may lead to neurological sequelae and even death [9].

Ambient heat exposure was suggested to be one of the most important environmental factors that were associated with an elevated risk of HFMD in numerous previous studies [10, 11]. For example, a systematic review on top of 11 studies previously conducted in Asia-Pacific regions suggested an average 5(2–8)% increase in the incidence of HFMD per 1°C increase in the temperature [12]. Similar findings were also observed in our previous research [13–16].

However, both literature and our previous findings were based on the reported number of cases solely, without considering the variation in severity of symptoms across cases. The severity of symptoms is an important indicator of the disease burden, with severer cases that may represent a stronger health impact of heat exposure and result in a higher disease burden. DALY is an established indicator of disease burden and has been widely used for the evaluation of the global and regional burden of diseases since the 1990s [17]. It was reported that the healthy life years (e.g., DALY) lost of a severe HFMD case was 2–4 times higher than that of a mild case [18]. Failing to differentiate cases of different severity or capture the true disease burden may lead to biased estimates of the health impact [19].

To address this important knowledge gap, we designed this study to estimate the daily disease burden (i.e. daily DALY) of HFMD at the city level by considering the severity of symptoms; and assess its potential association with the ambient temperature. We also evaluated how the association was modified by individual-level and city-level factors.

## 2. Methods

### 2.1 Ethics statement

This study was approved by the Institutional Review Board at the School of Public Health, Sun Yat-sen University (Approval number: 2021–018). All patient information of HFMD surveillance data were collected from China Center for Disease Control and Prevention (China CDC). No confidential information was included and all methods were carried out in accordance with relevant guidelines and regulations. All data employed in this study were deidentified prior to analysis and anonymized confidentiality, thus informed consent was not required.

### 2.2 Study population

This study covered the entire province of Guangdong, one of the largest provinces in South China with a total population exceeding 115 million as of 2019 [20]. We included all the HFMD cases reported to the surveillance system operated by China CDC during 2010–2012. Under the Infectious Diseases Act, the system covered both the clinically diagnosed cases which were defined by the National Clinic Guide as patients with the papular or vesicular rash on hands, feet, mouth, or buttocks, and laboratory-confirmed cases as clinically diagnosed cases with laboratory evidence of enterovirus infections [21]. All the cases were mandatorily reported with information including birth date, onset date, ZIP code for the residential address, and the severity (i.e., mild, severe, and fatal cases).

### 2.3 The outcome data

The primary outcome of this study was the disability-adjusted life year (DALY) which was estimated for each case and then summed up across cases reported for each day in each city. DALY is a measure of overall disease burden, expressed as the number of years lost due to ill-health, disability, or early death. Cases of different severity may be suffering from significantly different burdens of disease. Therefore, compared with the incidence or prevalence, DALY better captures the impact of environmental exposures. We calculated DALY with the following formula as used previously [22].


$$DALY=-\left[\frac{DCe^{-\beta a}}{{\left(\beta +r\right)}^{2}}\left[e^{-(\beta +r)L}(1+(\beta +r)(L+a))-(1+(\beta +r)a)\right]\right]$$

Where *D* is the severity weight used to measure the impacts of different severity on health. The severity weights for mild, severe, and fatal cases were set as 0.0285, 0.133, and 1 according to the acute episodes of infectious disease from GBD2013 [23]. *r* = 0.03 is the discount rate to convert the future benefits (e.g., healthy life years) into present-value terms. *Β* = 0.04 is the parameter from the age-weighting function regressing the wights on age to capture different social roles for the cases at different ages. *C* = 0.166 is the correction constant in the age-weighting function. *a* is the onset age of the case. *L* is the duration of disability (e.g., illness) or time lost due to premature mortality (i.e., death) which was set as 0.0252, 0.0361, and (74.71-a) years for the mild, severe, and fatal cases according to a prior survey [18]. Of which, 74.71 was the annual average life expectancy at birth from 2010 to 2012, in China [24]. For example, the DALYs for the mild, severe, or fatal cases of one year of age are 0.0001, 0.0008, and 33.6529, respectively. Since the DALY estimate for a fatal case was extremely high while the number of fatal cases was very limited for HFMD, we excluded the fatal cases from modeling to avoid irregular fluctuations and improve the reliability of estimation [25]. To assess the impact of fatal cases on the results, we included the fatal cases in the analysis and found that the overall trend was similar, but with very large fluctuations across cities (see S3 Fig).

### 2.4 Exposure and confounders

We obtained the observational data of weather at the monitoring site level from the China Meteorological Data Service Center (http://data.cma.cn/en), with one site for a city. Among the total 21 cities, Chaozhou and Foshan did not have any monitoring sites. Therefore, we assigned weather observations from the nearest sites (in Jieyang and Guangzhou, respectively) to these two cities. The primary exposure in this study was the daily average temperature (°C) at the city level.

Consistent with previous studies [26], we controlled for multiple confounders including relative humidity (%), day of the week, the indicator of holidays, and the potential time trend of case numbers. Humidity was another important weather factor that was suggested to be significantly associated with the risk of HFMD [27]. The indicators of weekday and holiday were used to fit the potential temporal pattern in the heat exposure and case reporting. Children may be more likely to expose to ambient heat during weekends and holidays while more likely to visit a doctor during workdays when they have symptoms. The time trend was commonly adjusted in time series analysis to remove the potential seasonality and long-term trends in the case series.

### 2.5 Study design and statistical analyses

We used a time series analysis design by matching the outcome, exposure, and confounders at the daily scale for each city. We used a two-stage framework to investigate the association between DALY and temperature.

In the first stage, we used a distributed lag non-linear model (DLNM) with a Gaussian link-function to estimate the cumulative change in DALY per Celsius increase in daily mean temperature (*β*, DALY/°C). The model was developed for each city and specified as follows:

$$DALY∼Gaussian(\mu_{DALY})$$

$$\mu_{DALY}=\alpha +\beta ∙cb(Temperature)+Humidity+DOW+Holiday+ns(Time,4/year)$$

Where *DALY* was the daily DALY at the city level, following a Gaussian distribution with *μ*_*DALY*_ as the expectation. *α* was the intercept. *β* was the regression coefficient of temperature. *cb*(*Temperature*) was the cross-basis (cb) function used to fit the association between DALY and temperature, as well as the variation of the association over the lag period. The lag was used to fit the incubation period from the infection to the onset of symptoms which usually was 14 days for HFMD [28]. We fit the association and its variation over the lag period with a natural cubic spline (ns) with 4 and 5 degrees of freedom (df), respectively. *DOW* was the day of the week (i.e., Monday, Tuesday, …, Sunday). *Holiday* was the indicator of holidays (i.e., 1 = holiday, 0 = non-holiday). *ns*(*Time*, *4/year*) was a natural cubic spline with 4 *df* per year (semi-annual seasonality) to fit the potential seasonality and long-time trend in the time series.

In this study, we used the *Akaike Information Criterion (AIC)* to determine the optimal combination of *df* for the splines from 2–5 and 3–8 for the association and its variation over the lag period, respectively. We used the generalized variance inflation factor (GVIF) [29] to evaluate the collinearity between the independent variables and confirm that the GVIFs for all factors in the model were below 10 [30]. Using Guangzhou, the capital city in the study area, as an example, we observed a GVIF of 1.129 for temperature, 1.201 for humidity, 1.006 for DOW, 1.063 for holiday, and 1.217 for the time trend, suggesting that collinearity was not an issue for this study.

In the second stage, we pooled the city-level temperature-DALY relationships to an overall association for the entire province using a random-effect multivariate meta-regression fitted through the restricted maximum likelihood (REML) [31]. In this stage, we also investigated how the city-level factors may have modified the association between DALY and temperature exposure. Specifically, we included the following potential effect modifiers respectively to a meta-regression model: population density, per capita GDP (gross domestic product), rate of municipal sewage treatment, garbage disposal rate, per capita green space in parks, passenger transporting rate, longitude, latitude, altitude, temperature, relative humidity, air pressure, rainfall, and sunshine hours. The multivariate extension of the likelihood ratio (*LR*) test was used to test the significance of meta-predictors and differences between models. The residual heterogeneity was measured and tested by the multivariate extension of *I*^*2*^ statistics and the Cochran *Q* test [31].

Furthermore, we estimated the DALY of HFMD that could be attributed to the excess exposure to temperature based on the overall DALY-temperature association developed in the second stage. Specifically, we used a forward perspective method [32] as follows:

$${AD}_{temperature,t}=[1-exp(-\sum_{l=0}^{L}\beta_{temperature,l})]∙\sum_{l=0}^{L}\frac{{DALY}_{t+l}}{L-l+1}$$

where *AD* is the attributed DALY; *DALY*_*t*_ is the DALY at time *t*; *l* = 0, 1,…, L, and *L* is the maximum lag; *β*_*temperature*,*l*_ is the contributions from the exposure (temperature) occurring at time *t* to the risk at times *t+l*.

Results were also stratified by individual characteristics including gender, age (<3yrs vs ≥3yrs), and regions (the Pearl River Delta region, East, West, North, as displayed in S1 Fig). Data cleaning and analyses were conducted using R software 4.1.1. The statistical tests were considered statistically significant when *P* < 0.05, on two sides.

## 3. Results

### 3.1 Data summary

**Fig 1:**
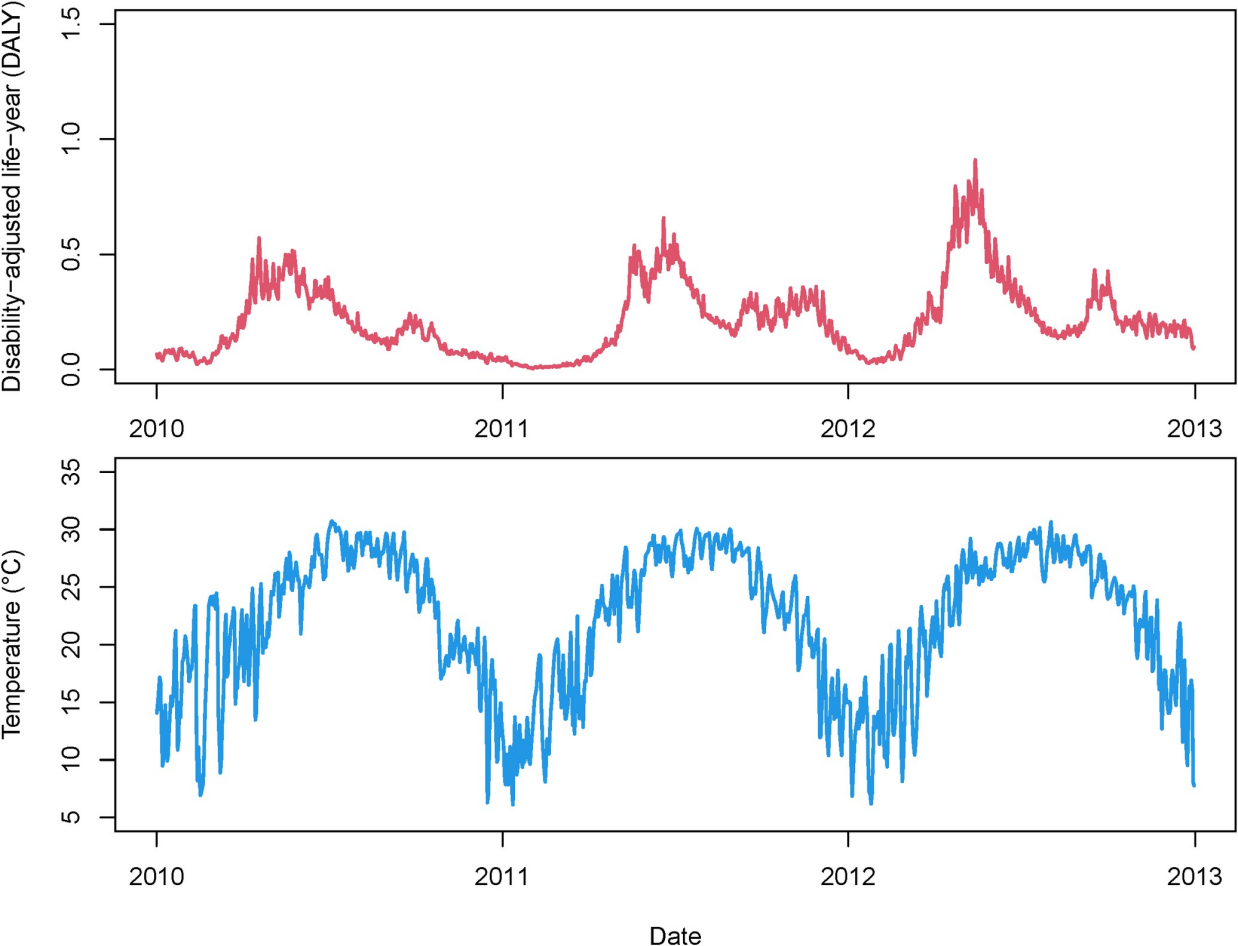
Daily time-series of DALY and average temperature of HFMD in Guangdong, 2010–2012. Daily DALY is the sum of the DALY of each day; Daily temperature is the average of the temperature of each day.

We observed that there were a total of 0.82 million cases reported in the study area during 2010–2012, with a total DALY of 4,451.161 years (Table 1). Most cases were male (64.24%) and under 3 years old (65.60%). We observed similar trends of DALYs and temperature over time (i.e. dates) in Fig 1, both with a major peak in the summer months of the year. However, we observed another minor peak in September following the major one for daily DALY. The Pearson’s correlation coefficient between daily DALY and temperature was 0.556 (*P*<0.001).

**Table 1:** Basic features of HFMD in Guangdong, 2010–2012.

	Total	2010	2011	2012
DALY	4451.161	2189.58	1245.378	1016.203
Total cases	830232	226175	273408	330649
Mild	826864(99.59)	224761(99.37)	272498(99.67)	329605(99.68)
Severe	3245(0.39)	1352(0.60)	876(0.32)	1017(0.31)
Fatal	123(0.01)	62(0.03)	34(0.01)	27(0.01)
Sex				
Male	533361(64.24)	146029(64.56)	176908(64.70)	210424(63.64)
Female	296871(35.76)	80146(35.44)	96500(35.30)	120225(36.36)
Age (year)				
<3	544653(65.60)	148199(65.52)	186434(68.19)	210020(63.52)
≥3	285579(34.40)	77976(34.48)	86974(31.81)	120629(36.48)
Region				
Pearl River Delta	585353(70.50)	159628(70.58)	188777(69.05)	236948(71.66)
East	60205(7.25)	14456(6.39)	24911(9.11)	20838(6.30)
West	63842(7.69)	17215(7.61)	16970(6.21)	29657(8.97)
North	120832(14.55)	34876(15.42)	42750(15.64)	43206(13.07)

HFMD: Hand, foot, and mouth disease; DALY: Disability adjusted life year.

Data were presented as numbers (proportions, %).

### 3.2 The association between DALY and temperature

**Fig 2:**
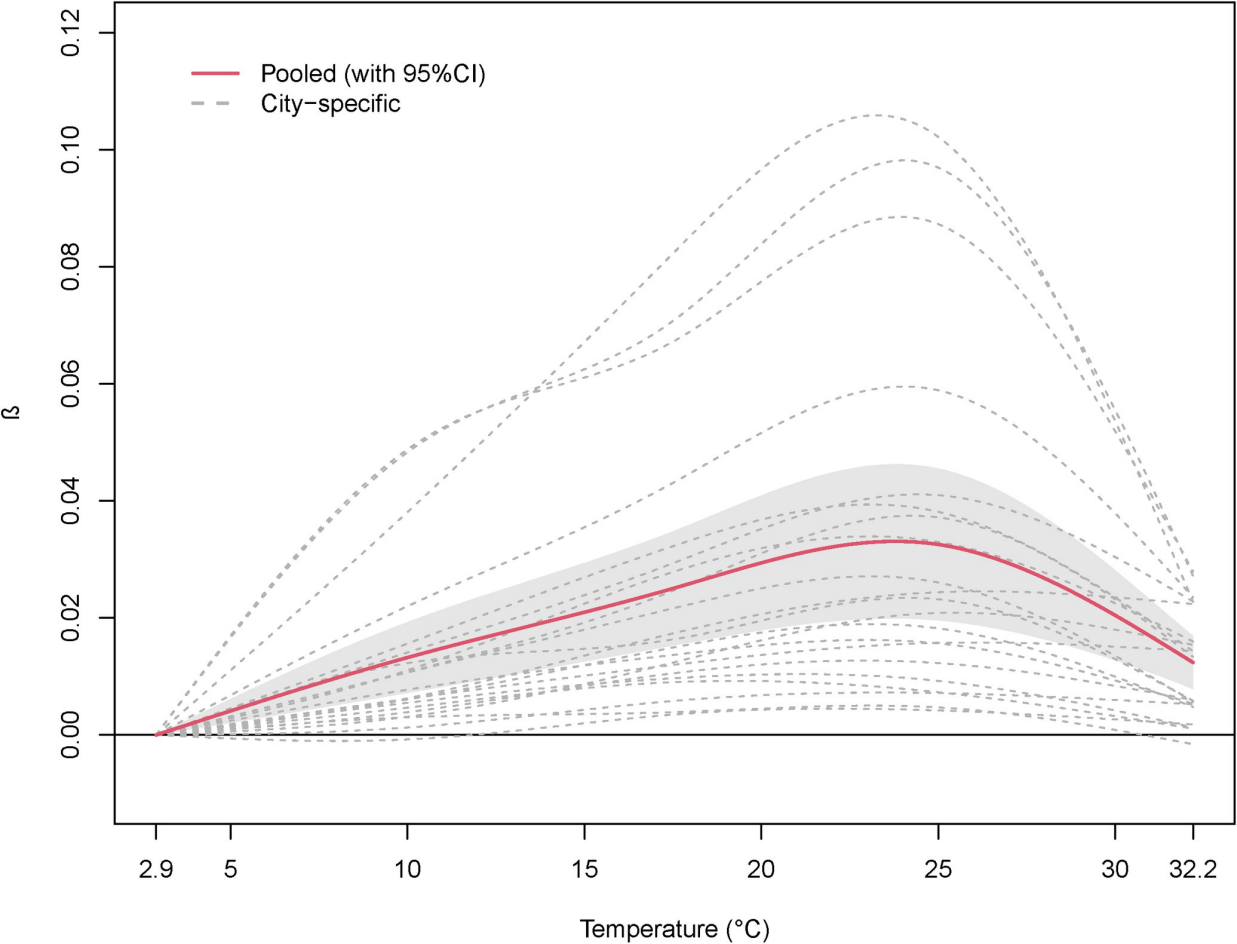
Overall cumulative predicted association of HFMD DALY associated with daily average temperature. HFMD: Hand, foot, and mouth disease; DALY: Disability-adjusted life year; Pooled: The results pooled the 21 city-specific results; *β* was the regression coefficient of temperature; The association was predicted on lag 0–14 days and reference temperature was 2.9°C.

We observed an inverse-U shape association between DALY and temperature, both for the city-level estimates and the pooled provincial estimates (Fig 2). Overall, the impact on DALY was significantly increasing with the daily average temperature, with the peak of heat effect identified at 23.8°C. We estimated that each degree increase in temperature at the peak may result in an increase of 0.0331 (95%CI: 0.0199–0.0463) DALYs lost, relative to the reference temperature (i.e., 2.9°C). Similarly, we estimated an increase of 0.0012 (95%CI: 0.0006, 0.0018) DALYs lost per degree increase in temperature at 3.5°C (the 2.5^th^ temperature), 0.0134 (95%CI: 0.0073, 0.0195) at 10.1°C (25^th^), 0.0250 (95%CI: 0.0153, 0.0348) at 17.5°C (50^th^), 0.0250 (95%CI: 0.0153, 0.0348) at 24.8°C (75^th^), and 0.0154 (95%CI: 0.0098, 0.0210) at 31.4°C (97.5^th^), as described in Table 2.

**Table 2:** Regression coefficients of temperature on HFMD DALY.

Subgroup	Temperature^#^, *β*(95%*CI*)					
	Max*	2.5^th^	25^th^	50^th^	75^th^	97.5^th^
Overall	0.0331(0.0199, 0.0463)	0.0012(0.0006, 0.0018)	0.0134(0.0073, 0.0195)	0.0250(0.0153, 0.0348)	0.0327(0.0197, 0.0458)	0.0154(0.0098, 0.0210)
Sex						
Male	0.0211(0.0128, 0.0295)	0.0008(0.0004, 0.0011)	0.0086(0.0047, 0.0125)	0.0159(0.0098, 0.0221)	0.0209(0.0127, 0.0291)	0.0098(0.0063, 0.0134)
Female	0.0119(0.0071, 0.0168)	0.0004(0.0002, 0.0006)	0.0048(0.0026, 0.0070)	0.0091(0.0055, 0.0127)	0.0118(0.0070, 0.0166)	0.0055(0.0034, 0.0077)
Age						
<3yrs	0.0126(0.0077, 0.0175)	0.0004(0.0002, 0.0007)	0.0049(0.0026, 0.0072)	0.0092(0.0058, 0.0126)	0.0125(0.0076, 0.0174)	0.0059(0.0039, 0.0080)
≥3yrs	0.0204(0.0122, 0.0287)	0.0007(0.0004, 0.0011)	0.0085(0.0047, 0.0122)	0.0158(0.0095, 0.0221)	0.0201(0.0120, 0.0283)	0.0094(0.0058, 0.0130)
Region						
Pearl River Delta	0.0591(0.0377, 0.0805)	0.0023(0.0012, 0.0034)	0.0254(0.0143, 0.0364)	0.0442(0.0277, 0.0606)	0.0585(0.0374, 0.0796)	0.0259(0.0175, 0.0344)
East	0.0100(0.0008, 0.0192)	0.0001(-0.0002, 0.0004)	0.0024(-0.0007, 0.0055)	0.0075(0.0011, 0.0140)	0.0100(0.0007, 0.0193)	0.0073(-0.0031, 0.0176)
West	0.0163(0.0112, 0.0214)	0.0003(0.0000, 0.0006)	0.0045(0.0021, 0.0069)	0.0116(0.0098, 0.0134)	0.0163(0.0112, 0.0214)	0.0126(0.0067, 0.0186)
North	0.0161(0.0096, 0.0226)	0.0006(0.0004, 0.0008)	0.0069(0.0048, 0.0090)	0.0137(0.0093, 0.0181)	0.0153(0.0086, 0.0221)	0.0052(0.0020, 0.0084)

HFMD: Hand, foot, and mouth disease; DALY: Disability adjusted life year.

*: Temperature with the maximum *β*; 23.8°C for overall, 23.8°C for male, 23.7°C for female, 24°C for age<3yrs, and 23.6°C for age≥3yrs; 23.9°C for Pearl River Delta, 24.2°C for East, 24.9°C for West, 22.5°C for North.

#: The temperature at 2.5th, 25th, 50th, 75th, and 97.5th are 3.5°C, 10.1°C, 17.5°C, 24.8°C, and 31.4°C, respectively.

**Fig 3:**
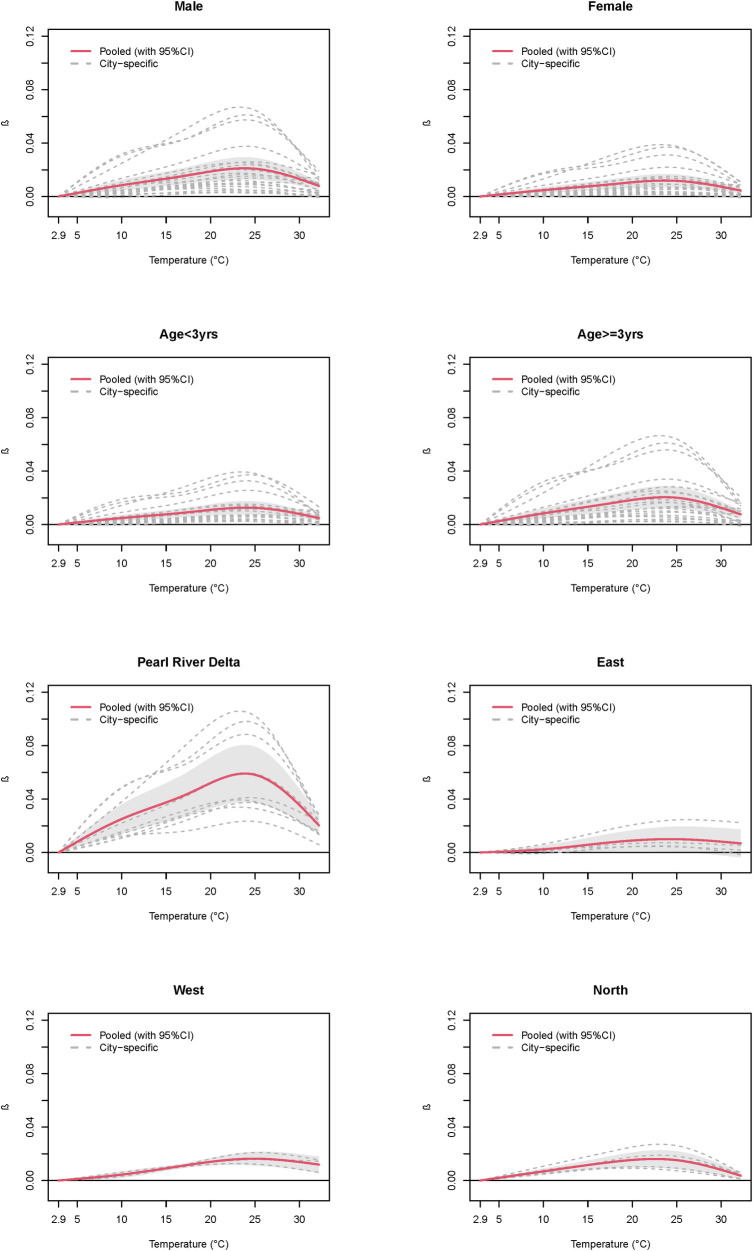
Overall cumulative predicted association of HFMD DALY associated with daily mean temperature by sex, age, and regions. HFMD: Hand, foot, and mouth disease; DALY: Disability adjusted life year. The association was predicted on lag 0–14 days and reference temperature was 2.9°C.

Similar associations were observed among subgroups by sex, age, and region (Table 2 and Fig 3). The peaks for males (23.8°C) and females (23.7°C) were observed at almost the same temperature, while the magnitude of the peak for males was slightly higher than that for females (*β*: 0.0211 vs 0.0119). The peak among children aged<3yrs (24°C) was observed at a slightly higher temperature than those aged ≥3yrs (23.6°C), while the magnitude of the peak among those aged ≥3yrs was greater (*β*: 0.0204 vs 0.0126). The peaks for different regions were observed at different temperatures (23.9°C for Pearl River Delta, 24.2°C for East, 24.9°C for West, and 22.5°C for North), and the magnitude of the peak in Pearl River Delta (*β* = 0.0591) was higher than those for the rest of regions (*β* = 0.01 for East, *β* = 0.0163 for West, *β* = 0.0161 for North).

### 3.3 The modification effect of city-level factors

The potential modification effect of city-level factors was reported in Table 3. Except for the passenger transporting rate (*P* value of *LR* test = 0.107), all other factors were observed with statistically significant modification effect in the meta-regression model with all *P* values of *LR* test < 0.001. We found that the per capita GDP had the least residual heterogeneity (*I*^2^) of 95.552% (*P* value of Cochran *Q* test < 0.001). The residual heterogeneity of the multivariate meta-regression model was 95.236% (*P* values of *LR* test and Cochran *Q* test < 0.001).

**Table 3:** Potential driven factors for the variance of DALY-temperature associations among cities.

Variables	*LR* test^a^	Cochran *Q* test^b^		*I*^2^(%)
		Statistic	*P* value	
Univariate model				
Population density	<0.001	3213.957	<0.001	97.635
per capita GDP	0.012	1708.775	<0.001	95.552
rate of municipal sewage treatment	<0.001	3419.458	<0.001	97.777
garbage disposal rate	<0.001	3452.952	<0.001	97.799
per capita green space in parks	<0.001	3285.539	<0.001	97.687
passenger transporting rate	0.107	2678.410	<0.001	97.162
longitude	<0.001	3106.724	<0.001	97.554
latitude	<0.001	3387.251	<0.001	97.756
altitude	<0.001	3108.362	<0.001	97.555
temperature	<0.001	3395.835	<0.001	97.762
relative humidity	<0.001	3445.789	<0.001	97.794
air pressure	<0.001	3159.569	<0.001	97.595
rainfall	<0.001	3389.415	<0.001	97.758
sunshine hours	<0.001	3039.460	<0.001	97.500
Multivariate model				
Without passenger transporting rate	<0.001	587.738	<0.001	95.236

^a^ *LR* test was used to test the significance of variables with the intercept-only model as reference.

^b^ Cochran *Q* test was used to test the significance of residual heterogeneity with the null hypothesis as no heterogeneity.

We further estimated the DALY-temperature relationship with the city-level factors specified at both low (25^th^) and high (75^th^) levels (S2 Fig), and found a significantly elevated impact of temperature on DALY in the areas with a higher population density, higher per capita GDP, or higher per capita green space in parks.

### 3.4 DALY attributed to the temperature exposure

With a maximum lag of 14 days, the attributable DALYs of HFMD due to temperature were estimated for all cases and subgroups by sex, age, and region (Table 4). Overall, a total of 6.432 (95%CI: 3.942–8.885) DALYs lost (attributable fraction, AF = 2.721%, 95%CI: 1.660–3.759%) could be attributed to temperature. Consistent with trends of association, an inverse-U shape relationship of attributable DALYs was observed. Specifically, we estimated the attributable DALYs as 0.046 (95%CI: 0.024–0.067) at the cold temperature and 0.680 (95%CI: 0.416, 0.932) at the heat temperature.

**Table 4:** Attribute DALYs of HFMD due to temperature in Guangdong, 2010–2012.

Subgroup	Temperature^#^, DALY(95%*CI*)		
	Overall	Cold	Heat
Overall	6.432 (3.924, 8.885)	0.046 (0.024, 0.067)	0.680 (0.416, 0.932)
Sex			
Male	2.632 (1.605, 3.637)	0.018 (0.010, 0.027)	0.281 (0.174, 0.387)
Female	0.839 (0.498, 1.173)	0.006 (0.003, 0.009)	0.088 (0.053, 0.123)
Age			
<3yrs	1.089 (0.670, 1.504)	0.007 (0.004, 0.011)	0.128 (0.080, 0.176)
≥3yrs	2.211 (1.319, 3.081)	0.016 (0.009, 0.024)	0.217 (0.131, 0.302)
Region			
Pearl River Delta	8.093 (5.188, 10.881)	0.057 (0.032, 0.084)	0.846 (0.561, 1.126)
East	0.129 (0.011, 0.247)	0.000 (0.000, 0.001)	0.017 (-0.002, 0.035)
West	0.226 (0.169, 0.281)	0.001 (0.000, 0.002)	0.028 (0.018, 0.038)
North	0.463 (0.288, 0.641)	0.004 (0.003, 0.006)	0.044 (0.023, 0.064)

HFMD: Hand, foot, and mouth disease; DALY: Disability adjusted life year.

#: The attributable components are separated in cold and heat by selecting as cut-off values the 10^th^ and 90^th^ percentiles of temperature distributions.

Regarding the intergroup comparison, similar disparities were observed as those in the association analysis (Table 4). We observed a higher attributable DALY among males (2.632, 95%CI: 1.605–3.637; relative to 0.839, 95%CI: 0.498–1.173 among the females), those aged ≥3yrs (2.211, 95%CI: 1.319–3.081; relative to 1.089, 95%CI: 0.67–1.504 among those aged <3yrs, and those in the the Pearl River Delta (8.093, 95%CI: 5.188–10.881; relative to 0.129, 95%CI: 0.011–0.247 among those from East; the 0.226, 95%CI: 0.169–0.281 among those from West; and 0.463, 95%CI: 0.288–0.641 among those from North).

## 4. Discussions

This study has unique advantages over previous research by accounting for the severity of symptoms and calculating the disease burden of HFMD cases. Although the health impact of ambient heat exposure was well documented, to the best of our knowledge, this is the first study designed to investigate the heat impact from the perspective of disease burden which more accurately captures the health impact than the traditional outcomes solely based on the reported number of cases.

DALY as an important indicator of disease burden was rarely used for infectious diseases. Among the limited studies focusing on the DALY associated with HFMD, Gan et al. (2015) estimated that the loss of DALYs for severe and mild HFMD cases were 3.47 and 1.76 person-year per 1000 persons, respectively [33]. Another multi-country study evaluated the burden of HFMD in eight high-burden countries (including China) in East and Southeast Asia and estimated that HFMD has been causing 96,900 (95% CI: 40,600–259,000) age-weighted DALYs annually [34]. However, these studies did not further link the burden of HFMD to environmental exposures which may have more public health implications.

We found an inverse-U shape relationship between the DALY of HFMD and the ambient temperature, with a peak of DALY identified at about 24°C. This finding indicated a monotonous increase in the disease burden of HFMD with temperatures under 24°C. Although limited evidence on the disease burden of HFMD was available for comparison, our finding was consistent with previous findings based on the incidence rate of HFMD. The association between heat exposure and the risk of HFMD has been well documented. For example, one of our previous studies suggested that each 1°C increase in temperature was associated with an increase of 1.86% (95% CI: 0.92, 2.81%) in the weekly number of HFMD cases among children under 14 years old [35]. The potential mechanism underlying the adverse impact of heat exposure remains unclear. However, the enhanced activity of HFMD associated virus and increased outdoor activities and exposures were considered the most plausible interpretations [14]. When the temperature continued to increase, we observed a declining trend in the estimated DALY of HFMD, which may be associated with reduced outdoor activities due to elevated heat exposure as well as increased use of air conditioning. Similarly, a study from the non-metropolitan areas of the Beijing-Tianjian-Hebei area also reported that the HFMD incidence no longer increased monotonically with the increasing temperature when the temperature exceeded 25°C [36]. In addition, we observed similar trends for the DALY estimates attributed to the temperature exposure.

According to our estimates, the timing (i.e. the temperature under which the maximum DALY was observed) of the DALY peak was generally consistent across subgroups, with the magnitude of the DALY at the peak being slightly greater among the male and those aged ≥3yrs and from the Pearl River Delta area. These findings were consistent with the previous findings. For example, Huang et al. reported that with the same temperature exposure, the risk of HFMD among the male tended to be greater than that among the female [37], which potentially resulted from the disparity in outdoor exposure frequency between boys and girls. Similarly, compared with children <3yrs, those ≥3yrs more likely to attend preschools, and have more outdoor activities and social contacts. Wang et al. observed that the *RR* estimates of temperature on the same-day HFMD incidence among children aged 3-5yrs were higher than that among those <1yr and 1-2yrs [38]. Regarding the geographical variation in the magnitude of peak DALY, our finding of the higher vulnerability among the children in the Pearl-river Delta region relative to other regions was consistent with our previous findings in the same area. We previously observed that the combined impact of temperature and humidity on the HFMD incidence tended to be higher in the Pearl River Delta than non-Pearl River Delta [21]. A potential possibility is that the Pearl River Delta region is much more prosperous, and children in this area might enjoy more outdoor activities with their family or friends during pleasant days. Furthermore, the Pearl-river Delta region was the most populated place in the study area, which may result in a much greater social contact among the population than in other regions. Similar trends across subgroups were observed for the attributable risk of temperature on HFMD DALY.

The geographical, climate, and socioeconomic factors were suggested to be potential effect modifiers for the DALY-temperature associations across different cities. Of those, the model with per capita GDP achieved the lowest residual heterogeneity. Similarly, Xiao et al. reported that in southern China with higher economic conditions, the temperature that corresponds to the highest epidemiological risk tended to be higher than that in northern China [39]. It is expected as the susceptible population from the areas with a higher level of per capita GDP and passenger transporting rate may have more social contacts. On the other side, the developed economy and established infrastructure in the developed areas are more likely to protect the urbanized population from the adverse impact of weather exposures [36]. Additionally, we found that the geographical and climate factors across cities may also modify the temperature-HFMD DALY association. The finding was consistent with a previous finding in the same study area which indicated that geographical factors such as latitude and longitude were important effect modifiers [40].

Some limitations of this study should be acknowledged. First, individual factors such as housing environment, smoking, drinking, and even educational levels may have significantly influenced the disease burden of HFMD. This study analyzed the data at the city level, which may have obscured some factors through the ecological fallacy effect. Second, because the daily number of cases for each community was insufficient for modeling or stratified analysis, all data were pooled at the city level for each day to achieve sufficient statistical power. Third, estimation of the association may be affected by excluding the fatal cases. However, this bias was more likely to underestimate the health impact of temperature towards the null.

## 5. Conclusions

Our study contributes to the limited knowledge on the association between temperature and the disease burden of HFMD epidemics. The moderate level of temperature was attributed to the more DALYs. Furthermore, the population density, per capita GDP, and per capita green space in parks may be the potential driven factors for the variation of DALY-temperature associations across different areas.

## Supporting information

S1 Fig Guangdong Province with four regions. The map was generated using R version 4.1.1 (https://www.r-project.org/) based on the boundary data from DIVA-GIS (http://www.diva-gis.org/).
(TIFF)

S2 Fig Estimated temperature-HFMD relationships by different levels of meta predictors. HFMD: Hand, foot, and mouth disease; Pooled: The results pooled the 21 city-specific results; *β* was the regression coefficient of temperature; The association was predicted on lag 0–14 days and reference temperature was 2.9°C.
(TIFF)

S3 Fig Overall cumulative predicted association of HFMD DALY associated with daily average temperature (including the fatal cases). HFMD: Hand, foot, and mouth disease; DALY: Disability-adjusted life year; Pooled: The results pooled the 21 city-specific results; *β* was the regression coefficient of temperature; The association was predicted on lag 0–14 days and reference temperature was 2.9°C.
(TIFF)

S4 Fig Estimated temperature-HFMD relationships by different virus types. HFMD: Hand, foot, and mouth disease; DALY: Disability-adjusted life year; Pooled: The results pooled the 21 city-specific results; *β* was the regression coefficient of temperature; The association was predicted on lag 0–14 days and reference temperature was 2.9°C.
(TIFF)
