# Supplementary figures and images for: Assessing the impact of ambient temperature on the risk of hand, foot, and mouth disease in Guangdong, China: New insight from the disease severity and burden
Source: PLoS Negl Trop Dis. 2022 Jun 23;16(6):e0010470. doi: 10.1371/journal.pntd.0010470 (PMC9223337; doi:10.1371/journal.pntd.0010470)

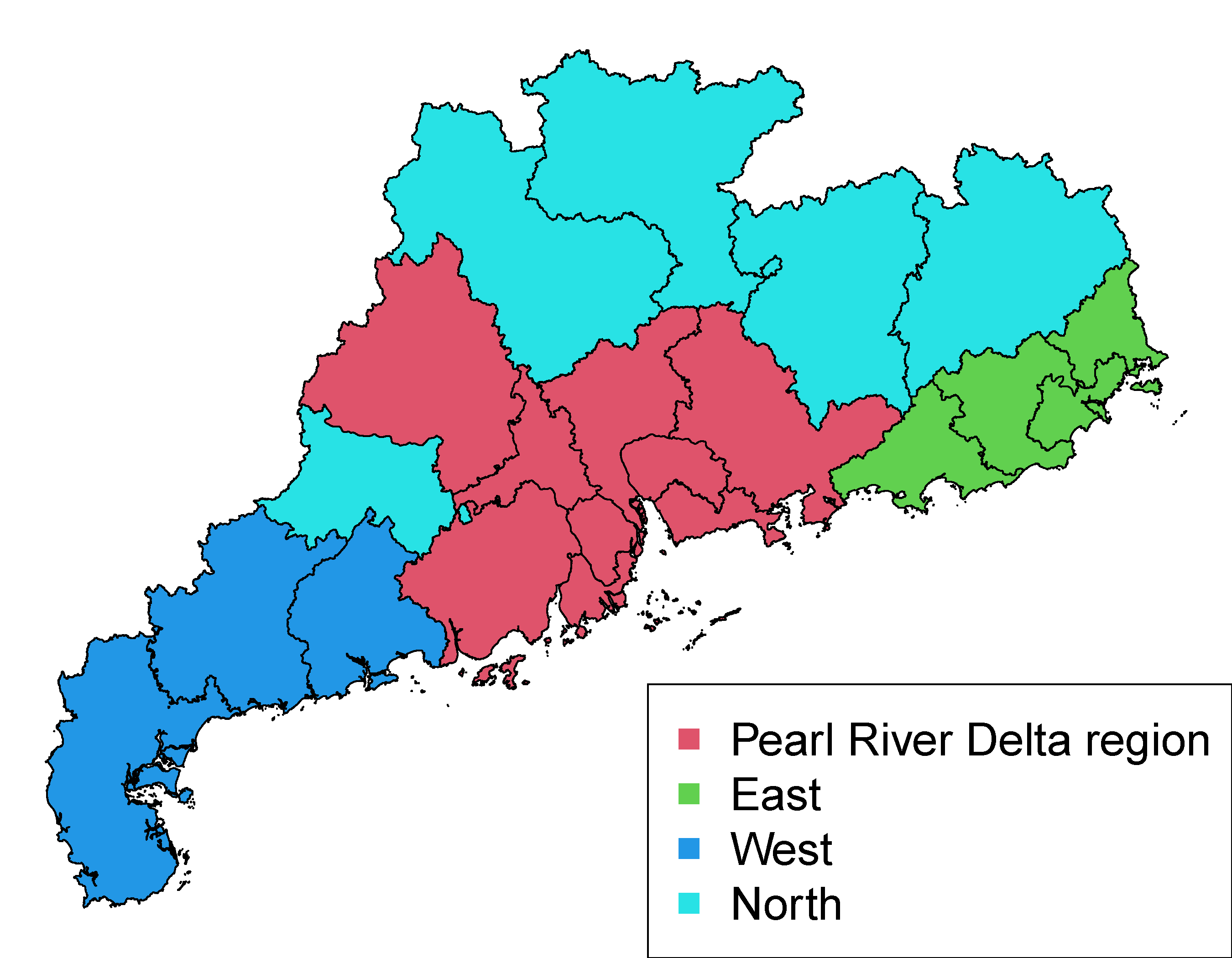

Supplement: S1 Fig — The map was generated using R version 4.1.1 (https://www.r-project.org/) based on the boundary data from DIVA-GIS (http://www.diva-gis.org/). (TIFF) [file pntd.0010470.s001.tiff]

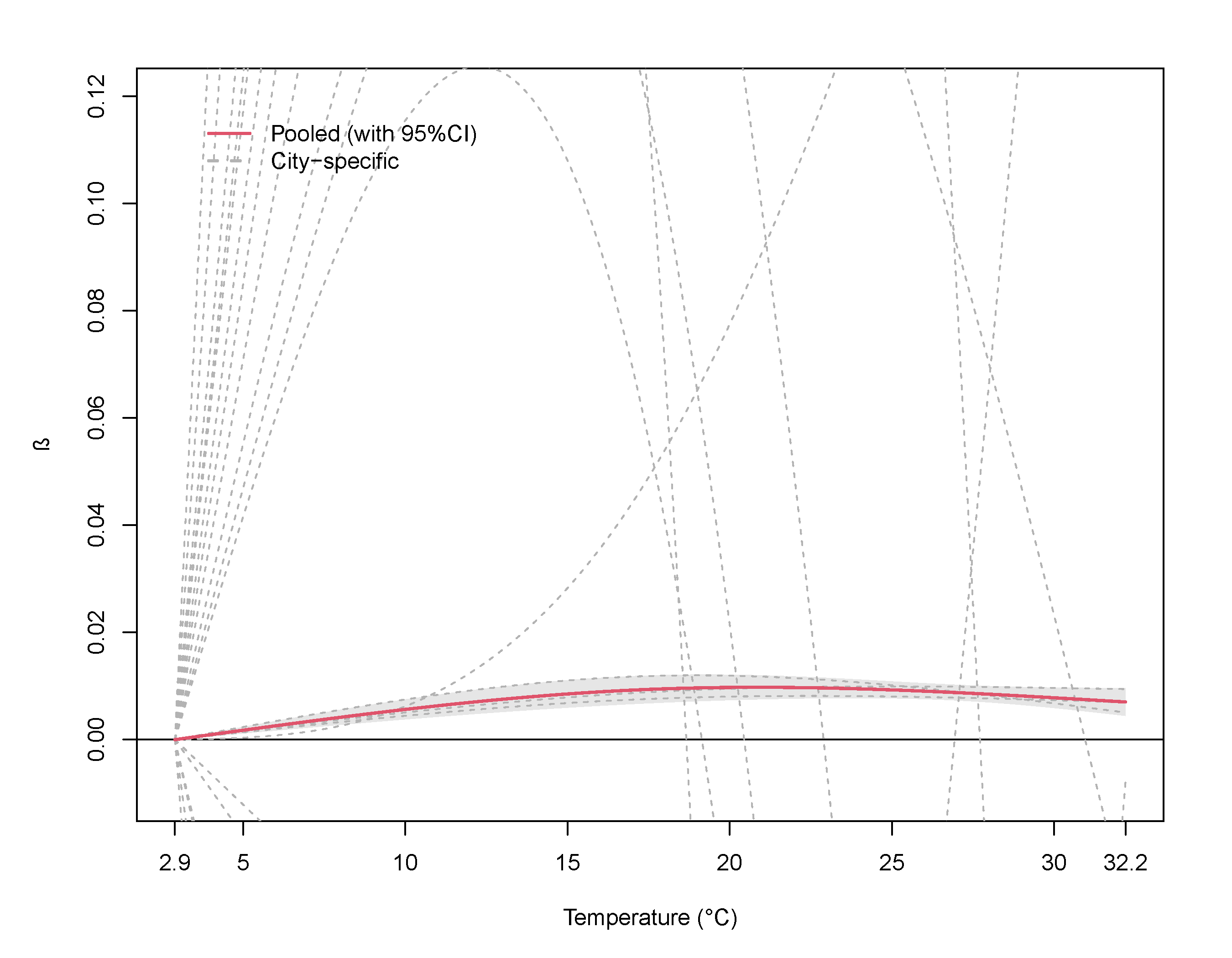

Supplement: S3 Fig — HFMD: Hand, foot, and mouth disease; DALY: Disability-adjusted life year; Pooled: The results pooled the 21 city-specific results; β was the regression coefficient of temperature; The association was predicted on lag 0–14 days and reference temperature was 2.9°C. (TIFF) [file pntd.0010470.s003.tiff]

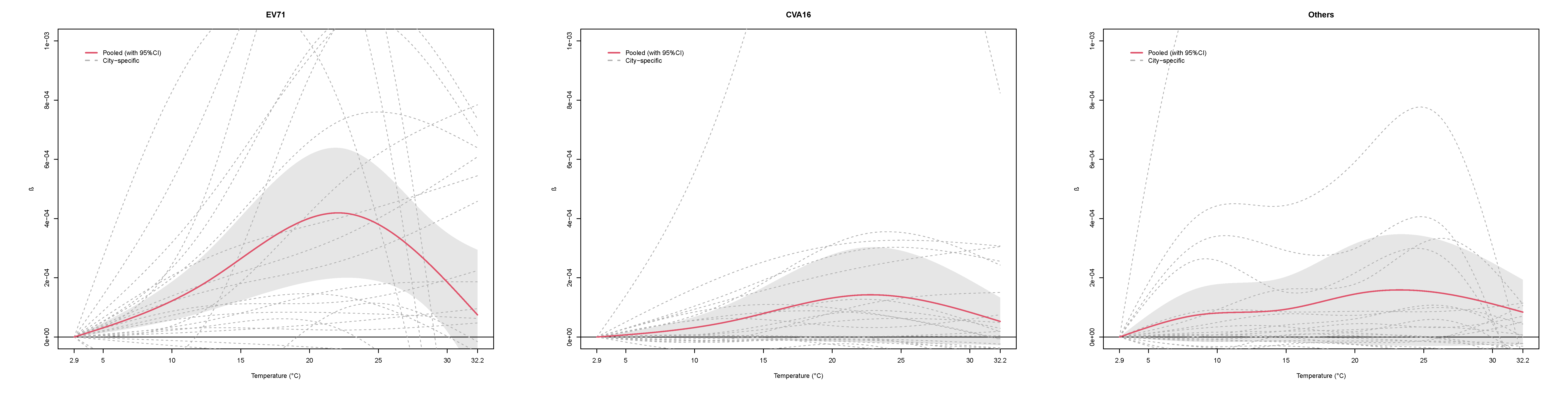

Supplement: S4 Fig — HFMD: Hand, foot, and mouth disease; DALY: Disability-adjusted life year; Pooled: The results pooled the 21 city-specific results; β was the regression coefficient of temperature; The association was predicted on lag 0–14 days and reference temperature was 2.9°C. (TIFF) [file pntd.0010470.s004.tiff]
